# Feasibility of a subcutaneous gluteal turnover flap without donor site scar for perineal closure after abdominoperineal resection for rectal cancer

**DOI:** 10.1007/s10151-019-02055-1

**Published:** 2019-08-20

**Authors:** R. D. Blok, J. A. W. Hagemans, J. W. A. Burger, J. Rothbarth, J. D. W. van der Bilt, O. Lapid, R. Hompes, P. J. Tanis

**Affiliations:** 1 Department of Surgery, Amsterdam University Medical Centres, University of Amsterdam, Meibergdreef 9, Post Box 22660, 1100 DD Amsterdam, The Netherlands; 2 LEXOR, Centre for Experimental and Molecular Medicine, Oncode Institute, Cancer Centre Amsterdam, Amsterdam UMC, University of Amsterdam, Meibergdreef 9, Amsterdam, The Netherlands; 3 Department of Surgical Oncology, Erasmus Medical Centre, Cancer Institute, Doctor Molewaterplein 40, Rotterdam, The Netherlands; 4 Department of Surgery, Catharina Hospital Eindhoven, Michelangelolaan 2, Eindhoven, The Netherlands; 5 Department of Surgery, Flevo Hospital, Hospitaalweg 1, Almere, The Netherlands; 6 Department of Plastic and Reconstructive Surgery, Amsterdam UMC, University of Amsterdam, Meibergdreef 9, Amsterdam, The Netherlands

**Keywords:** Rectal neoplasms, Abdominoperineal resection, Surgical flaps, Tissue transfer, Gluteal turnover flap, Perineal wound healing

## Abstract

**Background:**

Abdominoperineal resection (APR) carries a high risk of perineal wound morbidity. Perineal wound closure using autologous tissue flaps has been shown to be advantageous, but there is no consensus as to the optimal method. The aim of this study was to evaluate the feasibility of a novel gluteal turnover flap (GT-flap) without donor site scar for perineal closure after APR.

**Methods:**

Consecutive patients who underwent APR for primary or recurrent rectal cancer were included in a prospective non-randomised pilot study in two academic centres. Perineal reconstruction consisted of a unilateral subcutaneous GT-flap, followed by midline closure. Feasibility was defined as uncomplicated perineal wound healing at 30 days in at least five patients, and a maximum of two flap failures.

**Results:**

Out of 17 potentially eligible patients, 10 patients underwent APR with GT-flap-assisted perineal wound closure. Seven patients had pre-operative radiotherapy. Median-added theatre time was 38 min (range 35–44 min). Two patients developed a superficial perineal wound dehiscence, most likely because of the excessive width of the skin island. Two other patients developed purulent discharge and excessive serosanguinous discharge, respectively, resulting in four complicated wounds at 30 days. No flap failure occurred, and no radiological or surgical reinterventions were performed. Median length of hospital stay was 10 days (IQR 8–12 days).

**Conclusions:**

The GT-flap for routine perineal wound closure after APR seems feasible with limited additional theatre time, but success seems to depend on correct planning of the width of the flap. The potential for reducing perineal morbidity should be evaluated in a randomised controlled trial.

**Electronic supplementary material:**

The online version of this article (10.1007/s10151-019-02055-1) contains supplementary material, which is available to authorized users.

## Introduction

To date, abdominoperineal resection (APR) for low rectal cancer still carries a significant risk of perineal wound problems [1]. A recent randomised controlled trial on perineal wound closure after APR reported an incidence of complicated perineal wound healing of 34–37% at 30 days postoperatively [2], but perineal complications have even been reported to occur in up to 66% of patients after APR and primary closure [3]. In addition, patients may experience persisting perineal pain, or develop a chronic perineal sinus or perineal hernia [4–6].

The high risk of perineal morbidity after APR is related to the creation of a large pelvic dead space with bacterial contamination, making the surgical-site susceptible for infection. Furthermore, the use of pre-operative radiotherapy significantly impairs the healing capacity of this dead space—secondary to the decreased angiogenesis. To prevent these wound problems, immediate soft-tissue reconstruction has been advocated [7]. The rationale is that by obliterating the surgical dead space with well-vascularised tissues, the risk of wound breakdown and infection is reduced. Another reason is related to the concept that autologous tissue may add strength to the (partially) excised pelvic floor muscles, which may potentially reduce the risk of perineal hernia formation. There is, however, no consensus on the optimal method for perineal wound closure after APR.

Several techniques are used to improve perineal wound healing, including reconstruction using a V–Y fasciocutaneous flap, a vertical rectus abdominis myocutaneous (VRAM) flap, a gluteal, or a gracilis flap [8–11]. However, there are potential disadvantages to these techniques. These include the need for a plastic surgeon, a substantially increased theatre time and the potential for donor site and recipient site complications while often sacrificing the benefits of laparoscopy [12–17]. Moreover, as a large percentage of patients will not develop healing difficulties, immediate reconstruction with large muscle flaps might be an unnecessary risk and expense. An optimal harm–benefit ratio of reconstructive techniques is especially important in relatively low-risk patients undergoing non-extensive resections without pre-operative radiotherapy. There is an urgent need for a simple and minimally invasive technique for routine perineal wound closure after APR. We propose a novel unilateral subcutaneous gluteal turnover flap (GT-flap) without additional scarring or donor site morbidity.

The aim of this pilot study was to determine the feasibility of this procedure in patients who underwent APR for primary or recurrent rectal cancer.

## Materials and methods

### Design and patients

A prospective longitudinal multicentre interventional cohort study was performed in ten consecutive patients at two academic medical centres. All patients scheduled for extralevator APR were pre-operatively informed on the study in the outpatient clinics. Written informed consent was obtained for all participants. Inclusion criteria were adult patients (age ≥ 18 years) and planned for APR for primary or recurrent rectal cancer. Exclusion criteria were need for total pelvic exenteration, sacral resection above S4/S5, severe concomitant diseases affecting wound healing (i.e., renal failure requiring dialysis, liver cirrhosis, and peripheral vascular disease with Fontaine grade 3 or higher), or enrolment in other trials expected to influence perineal wound healing.

On day 7 and day 30 after surgery, the perineal wound was evaluated by residents or surgeons using the Southampton wound score (supplementary Table 1). Postoperative pain was assessed using the visual analogue scale (VAS) which ranged from 0 (no pain) to 10 (worst pain). In addition, photographs were taken, and all appointed wound scores were centrally reviewed by the trial coordinators (RDB and PJT). Patient demographics, neo-adjuvant treatment, tumour characteristics, and surgical details were intra-operatively collected. Postoperatively, type and extent of any wound event or any other adverse event, and all medical, radiological and surgical interventions were recorded to the last day of follow-up. The study protocol was approved by the Institutional Review Board of the Amsterdam UMC, University of Amsterdam, and the Erasmus MC (NL58380.018.16).

### Surgical procedure

All patients received antibiotic prophylaxis with Cefazolin and Metronidazole, with a repeat dose in case of prolonged surgery (> 4 h from time of first pre-operative dose). This was not extended postoperatively. APR started with skin incision close to the anus with subsequent dissection along the external sphincter, to preserve as much perineal skin and subcutaneous fat as possible without compromising oncologically safety.

**Fig. 1:**
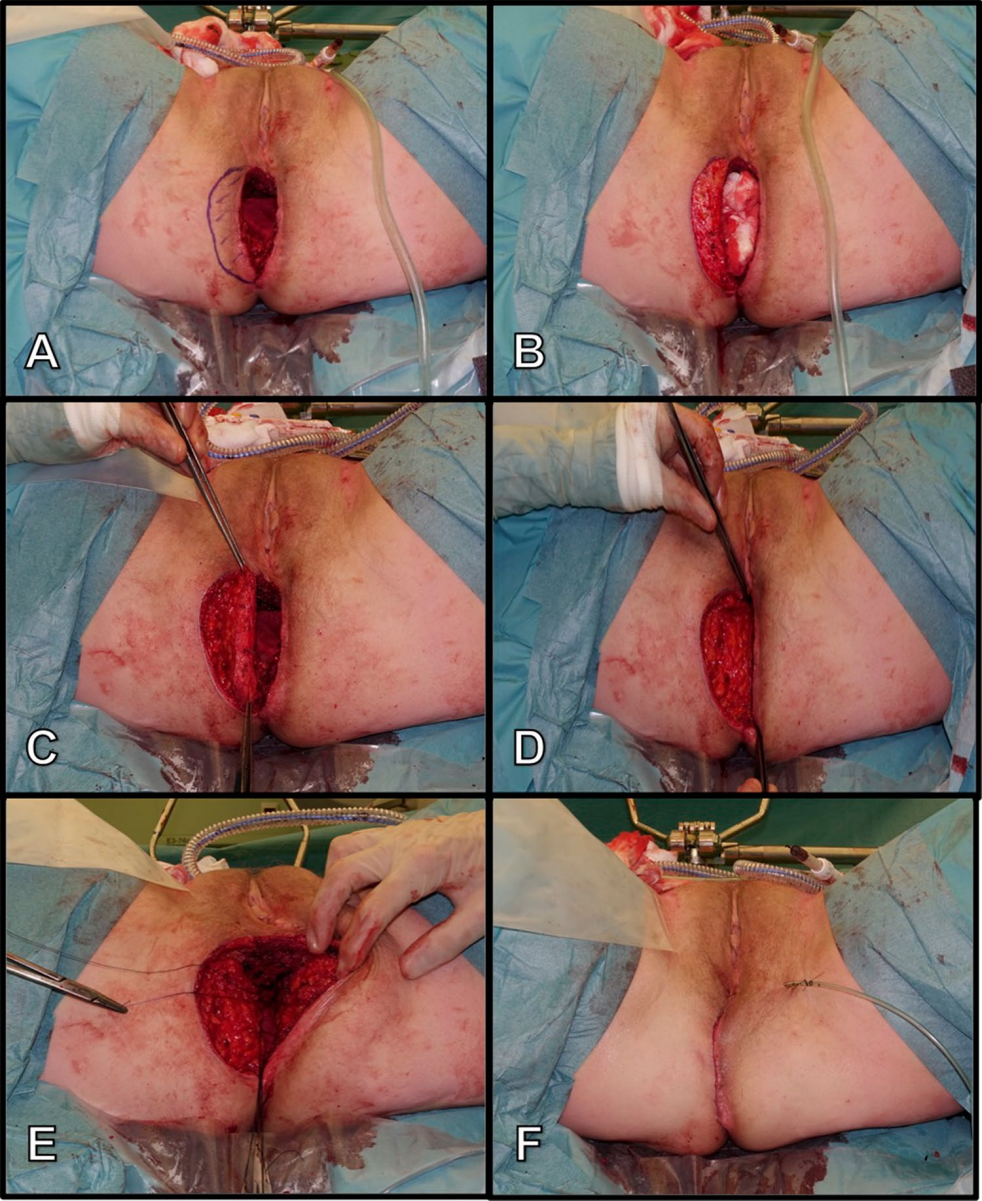
Reconstruction of an abdominoperineal defect using a gluteal turnover flap. **a** marking of the flap, **b** de-epithelialisation of the dermis, **c** flap after having transected onto the gluteal fascia, **d** rotation of the flap, **e** fixation of flap to the contra-lateral remnants of pelvic floor muscles, **f** midline scar following layered closure of the ischiorectal and perineal tissues over the flap

Perineal reconstruction is then performed using a unilateral, semilunar, de-epithelialised, subcutaneous GT-flap (Fig. 1). Creation of the GT-flap starts with drawing of a semi-circular incision adjacent to the surgical defect of approximately two-and-a-half centimetre in width. Success of the flap is contingent upon obtaining tension-free closure at the midline. For this reason, the flap can only be a few centimetres in width. Pre-operative mapping of the perforators is not deemed necessary due to the broad base of the turnover flap and its robust blood supply. Furthermore, due to the design of the flap, there is no need to elevate the flap on a single perforator. The flap is de-epithelialised followed by incision through the skin and slightly lateral dissection towards the gluteus muscle. It is important to perform the de-epithelialisation before dissecting the flap, as this makes it easier. The flap is then hinged into the defect, and the dermis is anchored to the contra-lateral remnants of the levator muscles. Next, a vacuum drain is positioned on top of the flap and the subcutaneous fat and perineal skin are closed in a layered fashion in the midline over the flap.

Patients were allowed to mobilise on the first postoperative day, but were instructed not to sit directly on the perineal wound for the first few days. The drain was removed after at least 3 days according to the surgeons’ judgement.

### Feasibility criteria and secondary outcome measures

The procedure was deemed feasible if (1) no more than five patients had a complicated wound healing at 30 days postoperatively and (2) including no more than two flap failures. Complicated wound healing was defined as a Southampton wound score equal or greater than II (supplementary Table 1). We hypothesised that, since the flap is covered and not visually accessible for evaluation of flap perfusion, flap failure would eventually result in wound breakdown. Therefore, flap failure was defined as a Southampton wound score of V.

Secondary endpoints were median length of the procedure, length of hospital stay, number of specific complications, and number of reinterventions and readmissions.

### Statistical analysis

Categorical data were expressed as absolute numbers with corresponding proportions and continuous data according to distribution as means with standard deviation (SD) or medians with interquartile range (IQR). The treatment effect was determined based on a per-protocol analysis. All analyses were performed with IBM SPSS statistics, version 24.0.0 (IBM Corp., Armonk, NY, USA).

## Results

Among 17 eligible patients, 11 were willing to participate and signed informed consent. Patient characteristics are demonstrated in Table 1. The mean age was 64 years (range 44–79 years) and 7 were male. Indications for APR were primary rectal cancer (*n* = 8), recurrent rectal cancer (*n* = 2), and one patient that had a clinical diagnosis of rectal cancer, but appeared to have recurrent prostate cancer on postoperative pathological examination. Pre-operative radiotherapy was given to eight patients.

**Table 1:** Patient characteristics

Patient	Age (years)	Sex	BMI (kg/m^2^)	ASA	Smoking	Diabetes	Prior pelvic surgery	Indication	Distance ARJ (cm)	Threatened MRF	Neo-adjuvant therapy
1	59	F	28.4	II	Never	Yes	Hysterectomy	Primary rectal cancer	1	Yes	Radio-chemotherapy
2	67	F	23.1	II	Never	No	None	Primary rectal cancer	3	Yes	Radio-chemotherapy
3	79	M	28.7	II	Never	No	None	Recurrent prostate cancer	NA	NA	None
4	48	M	31.8	II	Never	No	Transanal TME^NA^	Recurrent rectal cancer	0	NA	None
5	68	M	27.8	III	Never	No	None	Primary rectal cancer	4	Yes	Radio-chemotherapy
6^a^	71	M	27.93	III	Stopped > 10 years	No	Prostatectomy	Primary rectal cancer	0	Yes	Radio-chemotherapy
7	68	F	33.9	II	Never	No	None	Primary rectal cancer	0	Yes	Radio-chemotherapy
8	66	M	26.5	I	Stopped < 10 years	No	None	Primary rectal cancer	1	No	None
9	44	M	32.7	II	Stopped < 10 years	No	None	Primary rectal cancer	Missing	Yes	Radio-chemotherapy
10	73	F	30.0	III	Stopped > 10 years	Yes	Hysterectomy	Primary rectal cancer	Missing	Yes	Radio-chemotherapy
11	68	M	27.2	I	Stopped < 10 years	No	Laparoscopic TME	Recurrent rectal cancer	9	NA	Radio-chemotherapy

*BMI* body mass index, *ASA* American Society of Anaesthesiologists classification, *TME* total mesorectal excision, *ARJ* anorectal junction, *NA* not applicable, *MRF* mesorectal fascia

^a^Patient was excluded intra-operatively

Surgical details are shown in Table 2. In one case, it was not thought to be possible to obtain tension-free midline closure using a GT-flap due to the large size of the perineal skin defect after resection. A GT-flap with midline perineal closure could be performed in the remaining ten patients. Median total theatre time was 305 min (IQR 249–370 min), and median time taken for flap harvesting and insertion into the neo-pelvic floor was 38 min (IQR 35–44 min).

**Table 2:** Surgical details and intra-operative outcome

Patient	Type of APR	Position	Abdominal approach	Perineal approach	Adjacent organ resection	Intraop RTX	Omentoplasty	Abdominal drain	Type of surgeon	Buttock	Perineal drain	Skin closure	Reconstruction time^a^	Total theatre time^a^
1	Extralevator	Lithotomy	Open	Open	None	No	Yes	Yes	Plastic	Left	Yes	Transcutaneous	42	207
2	Extralevator	Lithotomy	Open	Open	None	No	Yes	Yes	Plastic	Left	Yes	Transcutaneous	55	510
3	Extralevator	Lithotomy	Open	Open	None	No	Yes	Yes	General	Left	Yes	Transcutaneous	45	302
4	Extralevator	Lithotomy	Laparoscopic	Open	None	No	No	Yes	Plastic	Left	Yes	Intracutaneous	35	151
5	Extralevator	Lithotomy	Open	Open	None	No	Yes	Yes	Plastic	Right	Yes	Transcutaneous	38	305
6	NA	NA	NA	NA	NA	NA	NA	NA	NA	NA	NA	NA	NA	NA
7	Extralevator	Lithotomy	Open	Open	None	No	Yes	Yes	General	Left	Yes	Transcutaneous	36	327
8	Extralevator	Prone	Laparoscopic	Open	None	No	No	Yes	Plastic	Right	Yes	Intracutaneous	Missing	Missing
9	Extralevator	Lithotomy	Laparoscopic	TAMIS	None	No	No	Yes	Plastic	Left	Yes	Intracutaneous	35	310
10	Extralevator	Lithotomy	Laparoscopic	TAMIS	Posterior vaginectomy	No	No	No	General	Left	Yes	Intracutaneous	31	291
11	Extralevator	Lithotomy	Open	Open	Left pelvic sidewall	Yes	Yes	Yes	Plastic	Right	No	Transcutaneous	40	412

*APR* abdominoperineal resection, *NA* not applicable, *TAMIS* transperineal minimally invasive surgery (using GelPOINT path and Airseal), *Intraop RXT* intra-operative radiotherapy

^a^Time in minutes

### Surgical outcome

**Fig. 2:**
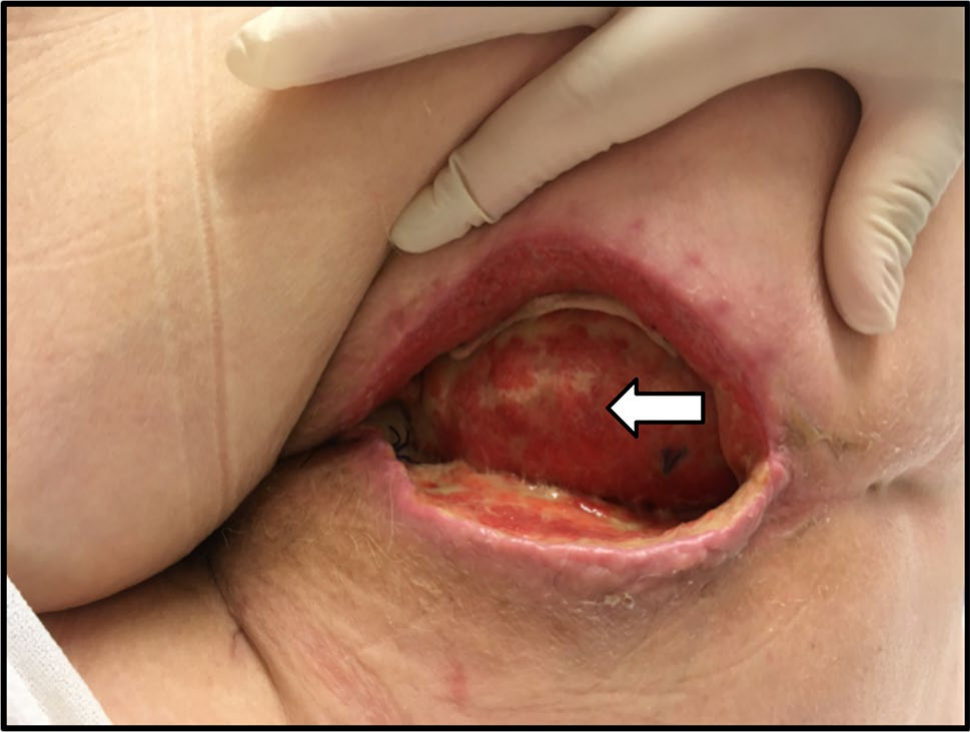
Perineal wound dehiscence 2.5 cm in depth with mild inflammation following abdominoperineal resection with insertion of gluteal turnover flap. The underlying subcutis of the flap is still viable (white arrow), and ensures that there is no atmospheric connection to the intra-abdominal cavity

Median length of follow-up was 33 days (IQR 27–35 days). Postoperative outcomes are displayed in Table 3. Median length of hospital stay was 10 days (IQR 8–12 days). In total, 4 patients had a complicated perineal wound healing at 30 days (Southampton wound score ≥ II). Two patients developed a superficial dehiscence of a few centimetres in depth, one with concomitant pus discharge. The underlying GT-flaps were unaffected (Fig. 2). Retrospective evaluation of these two patients revealed that the design of the flap was too wide, resulting in tension on the perineal wound after midline closure. Both patients needed no further treatment besides irrigation with saline. One patient developed perineal infection with a small pus pocket necessitating manual drainage and antibiotic therapy for 7 days. The last patient with a complicated wound healing at 30 days developed perineal pain secondary to a non-infected perineal seroma that required manual drainage in the outpatient clinic. There were no cases of flap necrosis. With a total of four complicated perineal wounds at 30 days and no flap failures, predefined feasibility criteria were met.

**Table 3:** Short-term outcome after abdominoperineal resection and gluteal turnover flap

Patient	Histopathology	Hospital stay (days)	7-day wound score^b^	30-day wound score^b^	VAS 7 days	VAS 30 days	Perineal complications	Perineal reinterventions	Other complications	Other interventions	Follow-up (days)
1	pT0N0Mx	8	0	0	0	0	None	None	UTI, urinary retention	AB for UTI	35
2	pT0N0Mx	10	I	IV	2	1	Infection	Manual drainage and AB	None	None	29
3	pT4N2Mx^a^	11	0	III	0	4	Dehiscence, Seroma	Manual drainage	None	None	36
4	pT3N0Mx	9	I	I	0	0	Seroma	Perineal irrigation	Ileus	TPN	41
5	pT3N0Mx	10	0	0	0	1	None	None	Ileus, urinary retention, abscess right buttock	I&D abscess	32
6	NA	NA	NA	NA	NA	NA	NA	NA	NA	NA	NA
7	pT3N0M1	13	IV	II	Missing	Missing	Dehiscence, Infection	None	UTI	AB for UTI	34
8	pT2N2M1	6	0	0	2	Missing	None	None	Urinary retention	Tamsulosine	20
9	pT0N0Mx	6	II	III	3	Missing	Seroma	Manual drainage	None	None	19
10	pT3N1Mx	20	III	0	Missing	Missing	Seroma	None	Ileus, pneumonia, delirium	TPN, AB for pneumonia, haldol	33
11	pT0N0Mx	8	0	0	Missing	3	None	None	Urinary retention	None	31

*NA* not applicable, *VAS* visual analogue pain scale (measured at rest), *AB* antibiotic therapy, *UTI* urinary tract infection, *TPN* total parenteral nutrition, *I&D* incision and drainage

^a^Recurrent prostate cancer

^b^According to the Southampton Wound Scoring System

Two more patients had perineal seroma, but both resolved within 30 days and without further treatment. In the remaining four patients, healing of the perineal wound was uneventful. Perineal pain at 7 days was reported for seven patients with a median VAS score of 0. Perineal pain at 30 days was reported for six patients with a median VAS score of 1. During follow-up, there were no readmissions, and no radiological or surgical reinterventions. Additional postoperative complications included ileus (*n* = 3), unrelated abscess on buttock (*n* = 1), urinary retention (*n* = 4), urinary tract infection (*n* = 2), pneumonia (*n* = 1), and delirium (*n* = 1).

## Discussion

This pilot study aimed to determine the feasibility of the GT-flap in routine perineal reconstruction after APR. The GT-flap was technically feasible with midline closure in all patients, except for one patient in whom more perineal skin had to be excised for oncological reasons. The flap added only limited additional theatre time. The majority of patients had uncomplicated perineal wound healing at 30 days postoperatively without any flap failure, thereby fulfilling our predefined feasibility criteria. Retrospective analysis of two cases of wound dehiscence revealed the critical part of the procedure, namely planning the appropriate width of the skin island that still allows for tension-free closure in the midline.

The GT-flap is only a valuable option if the perineal skin and subcutaneous fat can be maximally preserved from an oncological point of view. Therefore, distal rectal cancer without involvement of the perineal skin and subcutaneous fat requiring APR with a certain extent of resection of the levator muscles seems to be the optimal indication. If additional perineal skin has to be excised, for example, in case of advanced anal cancer or radiation-induced skin fibrosis, there is a need for flap-assisted closure that adds a skin island, such as the VRAM flap.

The GT-flap has a favourable profile, compared to existing literature on flap repair [7, 9]. There seems to be no partial necrosis of the flap or total flap loss, as can be observed with muscle flaps, although the sample size is still small [18]. The procedure can be combined with laparoscopy, contrary to conventional VRAM flaps for example. In addition, median additive theatre time was only 38 min, which included a learning curve and time needed for photographing the procedure. This is likely to decline in the future with increasing experience. Another benefit is the ease of flap harvesting, not necessarily requiring a plastic surgeon. Nonetheless, the procedure should preferably be performed by a surgeon already familiar with harvesting perforator flaps, or after initially being proctored by a plastic surgeon. Injury to the perforators can have serious consequences. If the flap is raised too large, this will lead to undue tension of the perineal skin, which can result in a major dehiscence. However, these basic principles of the technique are quite simple and easy to learn.

**Fig. 3:**
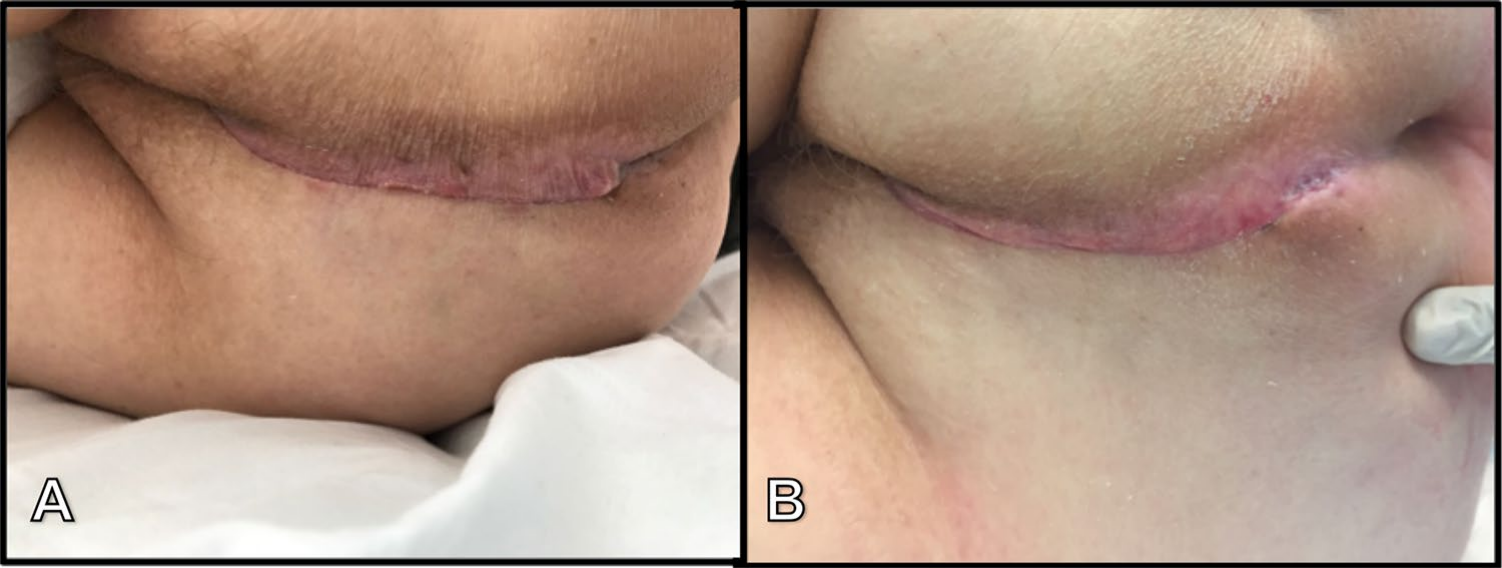
Healed perineal wound with symmetrical midline scarring **a** on day 7, and **b** day 30 after abdominoperineal resection and gluteal turnover flap for rectal cancer

Another advantage of the GT-flap over other options is the symmetrical midline scarring, thereby preserving the gluteal cleft and restoring normal perineal aesthetics (Fig. 3). This is a major advantage compared to the VRAM or conventional gluteal flaps (e.g., V–Y transposition, SGAP, IGAP), which leave both large and visible scarring. Furthermore, no dissection of muscle is performed. Patients are allowed to mobilise directly. The GT-flap seems to avoid problems with balance, sitting or walking secondary to muscle weakness or pain that is often seen after gluteal muscle transpositions. Considering these benefits, the GT-flap may be very attractive for routine perineal wound closure after APR.

In the current pilot study with its small sample size, we found no distinct improvement in perineal wound healing rate compared to recent literature on various closure techniques after APR [2, 7, 19]. In addition, when compared to outcomes from our institution (and two other hospitals) after APR with primary layered closure and pedicled omentoplasty, we observe similar wound healing rates [20]. However, this should be interpreted with caution, as this study was not powered to evaluate the efficacy of the GT-flap. Due to the study design, it was also not possible to assess the impact of the GT-flap on the risk of long-term perineal complications such as perineal hernia. It is our feeling that the flap may add strength to the neo-pelvic floor by anchoring the strong dermis to the contra-lateral remnants of the levator muscles. Future study should evaluate whether this provides sufficient support to potentially prevent perineal hernia in the long term. A recent publication by Chasapi et al. reported on a similar reconstructive procedure in 14 patients having APR for anorectal cancer [21]. The type of flap used differed from the technique described here in that the flap was detached from the gluteal fascia with one remaining perforator for blood supply. They showed favourable outcome with only 1 patient suffering from superficial skin dehiscence, and 1 developing a perineal hernia 7 months after surgery. These findings support our feeling that in selected patients, adjacent gluteal skin and subcutaneous fat can be relatively easily used for perineal closure after APR, with the potential advantages of reduced perineal morbidity by filling the space of the resected anal sphincter complex. The next step is conducting a randomised controlled trial to determine the effectiveness of this intervention in reducing perineal morbidity after APR, both short term and long term, comparing GT-flap with other flaps or other techniques for perineal closure reporting promising outcomes, such as biomesh [22].

## Conclusions

The GT-flap is a technically feasible and safe method for perineal wound closure after APR in patients with primary or recurrent rectal cancer if no additional perineal skin has to be sacrificed. The procedure is relatively quick and easily applicable, and seems associated with no apparent donor site morbidity or scarring. Further research is warranted to assess the potential for reducing perineal wound morbidity and to evaluate long-term quality of life.

## Electronic supplementary material

Below is the link to the electronic supplementary material.

Supplementary material 1 (PDF 87 kb)
