# Supplementary material for: Feasibility of a subcutaneous gluteal turnover flap without donor site scar for perineal closure after abdominoperineal resection for rectal cancer
Source: Tech Coloproctol. 2019 Aug 20;23(8):751–9. doi: 10.1007/s10151-019-02055-1 (PMC6736901; doi:10.1007/s10151-019-02055-1)
Supplement: Supplementary file 1 — Supplementary material 1 (PDF 87 kb) [file 10151_2019_2055_MOESM1_ESM.pdf]

Article title: Feasibility of a subcutaneous gluteal turnover flap without donor site scar for perineal closure after abdominoperineal resection for rectal cancer

Journal name: Techniques in Coloproctology

Author names: R.D. Blok, J.A.W. Hagemans, J.W.A. Burger, J. Rothbarth, J.D.W. van der Bilt, O. Lapid, R. Hompes, P.J. Tanis

Corresponding author: P.J. Tanis, M.D., Ph.D.; Department of Surgery, Amsterdam University Medical Centres, University of Amsterdam, Post box 22660, 1100 DD Amsterdam, the Netherlands; Phone: +31 20 566 9111; Fax: +31 20 566 6569; E-mail: P.J.Tanis@amc.nl.

Supplementary table 1. Southampton wound scoring system

Grade	Appearance
**0**	Normal healing
**I Normal healing with mild bruising or erythema:**	
A	Some bruising
B	Considerable bruising
C	Mild erythema
**II Erythema plus other signs of inflammation:**	
A	At one point
B	Around sutures
C	Along wound
D	Around wound
**III Clear or haemoserous discharge:**	
A	At one point only (<2cm)
B	Along wound (>2cm)
C	Large volume
D	Prolonged (>3 days)
**IV Pus:**	
A	At one point only (<2cm)
B	Along wound (>2cm)
**V Deep or severe wound infection with or without tissue breakdown; haematoma requiring aspiration**	
